# Assessing the Nature of Lipid Raft Membranes

**DOI:** 10.1371/journal.pcbi.0030034

**Published:** 2007-02-23

**Authors:** Perttu S Niemelä, Samuli Ollila, Marja T Hyvönen, Mikko Karttunen, Ilpo Vattulainen

**Affiliations:** 1 Laboratory of Physics, Helsinki University of Technology, Helsinki, Finland; 4 Department of Applied Mathematics, The University of Western Ontario, London, Ontario, Canada; Harvard University, United States of America; 2 Helsinki Institute of Physics, Helsinki University of Technology, Helsinki, Finland; 3 Wihuri Research Institute, Helsinki, Finland; 5 Memphys Center for Biomembrane Physics, University of Southern Denmark, Odense, Denmark; 6 Physics Department, University of Southern Denmark, Odense, Denmark; 7 Institute of Physics, Tampere University of Technology, Tampere, Finland

## Abstract

The paradigm of biological membranes has recently gone through a major update. Instead of being fluid and homogeneous, recent studies suggest that membranes are characterized by transient domains with varying fluidity. In particular, a number of experimental studies have revealed the existence of highly ordered lateral domains rich in sphingomyelin and cholesterol (CHOL). These domains, called functional lipid rafts, have been suggested to take part in a variety of dynamic cellular processes such as membrane trafficking, signal transduction, and regulation of the activity of membrane proteins. However, despite the proposed importance of these domains, their properties, and even the precise nature of the lipid phases, have remained open issues mainly because the associated short time and length scales have posed a major challenge to experiments. In this work, we employ extensive atom-scale simulations to elucidate the properties of ternary raft mixtures with CHOL, palmitoylsphingomyelin (PSM), and palmitoyloleoylphosphatidylcholine. We simulate two bilayers of 1,024 lipids for 100 ns in the liquid-ordered phase and one system of the same size in the liquid-disordered phase. The studies provide evidence that the presence of PSM and CHOL in raft-like membranes leads to strongly packed and rigid bilayers. We also find that the simulated raft bilayers are characterized by nanoscale lateral heterogeneity, though the slow lateral diffusion renders the interpretation of the observed lateral heterogeneity more difficult. The findings reveal aspects of the role of favored (specific) lipid–lipid interactions within rafts and clarify the prominent role of CHOL in altering the properties of the membrane locally in its neighborhood. Also, we show that the presence of PSM and CHOL in rafts leads to intriguing lateral pressure profiles that are distinctly different from corresponding profiles in nonraft-like membranes. The results propose that the functioning of certain classes of membrane proteins is regulated by changes in the lateral pressure profile, which can be altered by a change in lipid content.

## Author Summary

Biological membranes are complex 2-D assemblies of various lipid species and membrane proteins. For long, it was thought that the main role of lipid membranes is to provide a homogeneous, liquid-like platform for membrane proteins to carry out their functions as they diffuse freely in the membrane plane. Recently, that view has changed. It has become evident that several lipid environments with different physical properties may coexist, and that the properties of the different lipid domains may play an active role in regulating the conformational state and dynamic sorting of membrane proteins. We have carried out atom-scale computer simulations for three-component lipid bilayers, so-called lipid rafts, rich in cholesterol and sphingolipids. They show that arising from the local interactions between the lipid species, the elastic and dynamic properties of the membranes depend strongly on the lipid composition. The changes in elastic properties are suggested to alter the functional states of various membrane proteins. Changes in lipid composition are also shown to alter the distribution of local pressure inside the membrane. This is likely to affect proteins that undergo large anisotropic conformational changes between the functional states, such as the ion channel MscL, used as an example here. A great number of important physiological phenomena, such as transmitting neural impulses or trafficking molecules in and out of the cell, involve activation of membrane proteins, so it is relevant to understand all factors affecting them. Our findings support the idea that general physical properties of the lipid environment are capable of regulating membrane proteins.

## Introduction

The understanding of lipid membrane structures and their role in cellular functions has developed significantly since the introduction of the classical fluid-mosaic model by Singer and Nicolson [1]. The fluid-mosaic model predicted that cellular membranes are fluid and characterized by random distribution of molecular components in the membrane, resulting in lateral and rotational freedom. The more recent picture is considerably more elaborate, however. A large number of experimental results converge toward the idea that lateral domains enriched in sphingomyelin (SM) and cholesterol (CHOL) exist in biological membranes. These nanosized domains, called functional lipid rafts, have been suggested to take part in various dynamic cellular processes such as membrane trafficking, signal transduction, and regulation of the activity of membrane proteins [2–4]. The existence of stable lipid rafts in biological membranes is under intense scrutiny, and their existence is actually under debate since the lipid rafts, if they do exist, are probably too small to be resolved by techniques such as fluorescence microscopy [5]. Direct evidence of rafts in vivo is mainly based on monitoring the motions of membrane proteins [6] or on differential partitioning of fluorescent probes in membrane environments [7]. It is, however, difficult to perform experiments using living cells, which complicates measurements of physical quantities of the rafts, such as the exact lipid composition, characteristic size, and lifetime [8,9]. In model membranes, the coexistence of domains in the liquid ordered (*l_o_*) and the liquid disordered (*l_d_*) phase is widely accepted [9,10]. For example, the *l_d_* phase may be formed by an unsaturated phosphatidylcholine (PC), while the formation of the *l_o_* phase is promoted by a mixture of SM and CHOL. As for rafts, the current understanding of lipid rafts in biological membranes suggests a granular structure of nanometer-scale domains of various compositions [9,11,12] rather than a large-scale phase separation.

The exact nature of the underlying interactions that lead to lipid immiscibilities in membranes is under debate [13,14]. CHOL is particularly important as it has been shown to increase the conformational order of acyl chains and reduce the bilayer area, hence significantly increasing the packing density of the lipids [15–17]. CHOL is particularly effective in reducing the void space within the acyl chain region of the lipids [15], which is related to suppressed area compressibility and increased bending rigidity of the membrane with increasing CHOL concentrations. However, the lateral diffusion rates are not expected to slow down by more than a factor of 2–3 when the *l_d_* phase is compared with CHOL-induced *l_o_* phase [6,18]. Also, CHOL has recently been reported to significantly alter the lateral pressure profile of membranes [19]. This is important, as changes in the lateral pressure profiles have been suggested to be related to changes in membrane protein structure and activity [20].

Considering that the smallest estimates for the sizes of rafts fall in the range of nanometers [21,22], they make an accessible subject for computational studies. Though, in spite of the considerable importance of rafts, it is somewhat surprising that only a few atom-scale simulations have dealt with ternary mixtures of CHOL, SM, and PC [23,24], concentrating mainly on small-scale structural properties and local interactions between the lipids. In particular, there are no previous atom-level computational studies of rafts aiming to characterize the nature of their structural and dynamical features. For example, the nanometer scale structure within raft domains and its interplay with CHOL-induced effects are not understood. Further, the resulting large-scale properties, such as membrane elasticity in ternary raft-like lipid mixtures, are not understood either. Finally, and perhaps most importantly, the lateral pressure profiles associated with rafts are completely unknown. The concept of the lateral pressure profile across the lipid membrane is exceptionally significant, since it describes the pressure exerted on molecules embedded in a membrane. Cantor has proposed that incorporation of molecules into membrane and changes in lipid content would alter the lateral pressure profile across a membrane, and hence changes in the pressure profile would induce changes in membrane protein structure [20,25]. Experimental studies of this issue are remarkably difficult, however: currently there is only one study that employed fluorescent probes to gauge the overall shape of the lateral pressure profile [26]. Evidently, detailed atomistic simulations are called for.

**Figure 1:**
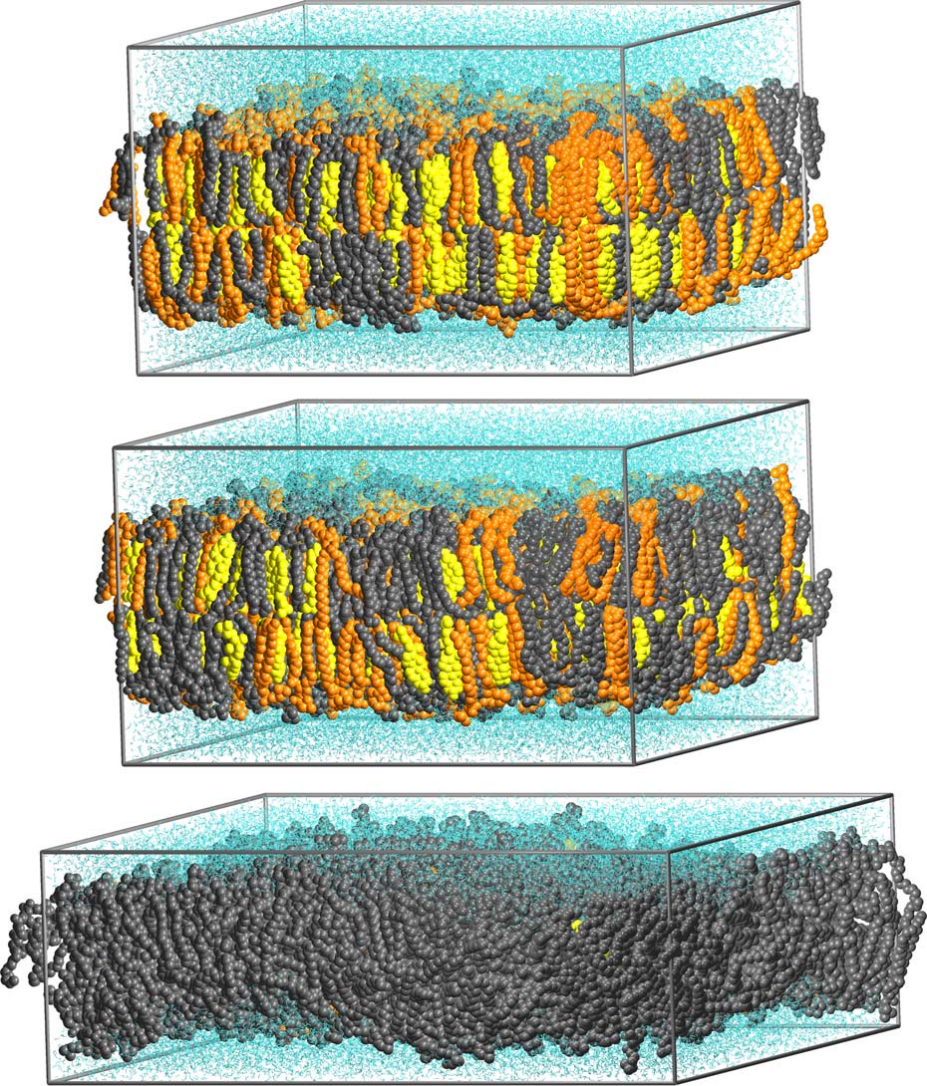
Snapshots at the End of Simulations for Systems *S_A_* (Top), *S_B_* (Middle), and *S_C_* (Bottom) POPC molecules are shown in gray, PSM in orange, CHOL in yellow, and water in cyan.

The state-of-the-art extent of the simulations conducted in this work, 15–20 nm in lateral dimensions and 100 ns in time, enables a reliable quantitative analysis of the properties of raft-like membranes not accomplished before. We employ large-scale atom level simulations for three mixtures of palmitoyloleoylphosphatidylcholine (POPC), PSM, and CHOL. The molar fractions are POPC:PSM:CHOL = 1:1:1, 2:1:1, and 62:1:1 for systems that we call *S_A_, S_B_,* and *S_C_,* respectively (see Figure 1). Based on a recent experimental phase diagram [27], these mixtures are expected to display the coexistent *l_o_* and *l_d_* phase domains (*S_A_* and *S_B_*) or a single *l_d_* phase (*S_C_*). Here, we illustrate the distinct nature of raft-like domains in three parts. First, we consider the elastic, thermodynamic, and dynamic properties of rafts that turn out to be very different from those of nonraft-like membranes. Second, we provide evidence that the presence of PSM and CHOL in raft-like membranes leads to strongly packed and rigid bilayers, characterized by significant nanoscale lateral heterogeneity within the raft domains. These findings express the prominent role of favored lipid–lipid interactions within rafts and highlight the significant role of CHOL in promoting the formation of rafts. Third, we provide compelling evidence that the lateral pressure profiles can be altered by a change in lipid content. In particular, we show how the presence of PSM and CHOL leads to intriguing lateral pressure profiles that are distinctly different from corresponding lateral pressure profiles in nonraft-like membranes, proposing that lipid membranes may regulate the functioning of certain classes of membrane proteins such as mechanosensitive channels through changes in lipid composition, and hence the lateral pressure profile.

## Results

### Elastic, Thermodynamic, and Dynamic Properties

Selected properties of the simulated membranes are summarized in Table 1. For system *S_C_,* the average area per lipid, *A,* and the bilayer thickness, *d,* are in agreement with previous findings on pure POPC bilayers [28,29], indicating negligible effects of PSM and CHOL on the bilayer dimensions. Also, the area compressibility modulus, *K_A_,* and the bending rigidity, *k_c_,* are in line with previous studies of pure PC bilayers, reporting *K_A_* = 140–300 × 10^−3^ N/m and *k_c_* = 4–9 × 10^−20^ J [30–32]. The lateral diffusion coefficient, *D,* for POPC in system *S_C_* is about 50% lower than the value of 1.4 × 10^−7^ cm^2^/s measured for pure POPC bilayer at 313 K [33]. A similar trend was found in comparison of our previous simulations on pure SM and PC bilayers [34] with this particular study [33]. This suggests that bilayer *S_C_* is close to the liquid disordered state of a POPC bilayer. This is also supported by the finding that small CHOL [33] or SM [35] concentrations have minor effects on *D* values of PC above melting temperatures.

**Table 1:**
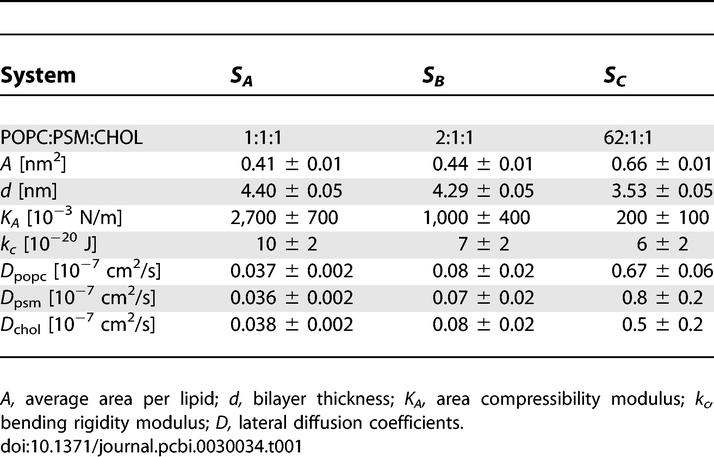
Average Structural and Thermodynamic Properties Calculated from the Simulations of Systems *S_A_*, *S_B_,* and *S_C_*

The condensing effect of CHOL becomes evident when comparing the values of *A* and *d* between systems *S_A_* to *S_C_*. As suggested in several previous works, CHOL's tendency to increase the order of neighboring acyl chains leads to decreased area per lipid and increased bilayer thickness upon increasing CHOL concentration [36–38]. Comparison with previous studies shows that the values for *A* in Table 1 for *S_A_* and *S_B_* are 0.1–0.4 nm^2^ lower than expected for binary PC–CHOL systems with similar CHOL concentrations [15,16]. CHOL's strong tendency to reduce fluctuations and increase the rigidity of membranes is best revealed by the *K_A_* values in Table 1. Previous reports have predicted maximally 5-fold to 7-fold increases in the *K_A_* values upon CHOL addition into PC bilayers [16,39]. The presently found unexpectedly large *K_A_* and small *A* as compared with PC–CHOL systems suggests an additional effect of PSM to decrease area fluctuations, possibly related to the tendency of SM to form intermolecular hydrogen bonds [34,40]. This idea is further supported by an experiment reporting a much higher value of *K_A_* (1,718 × 10^−3^ N/m) for a SM–CHOL bilayer than the value for a PC–CHOL bilayer (*K_A_* = 781 × 10^−3^ N/m), both with 50 mol% CHOL [41]. Our values for the bending rigidity, *k_c_,* are roughly in line with experimental results for PC–CHOL mixtures, which have shown a 120%–170% increase in the *k_c_* value upon increasing CHOL fraction from 0 to 30–50 mol% [31,42]. As the experimental values vary and computational reports on CHOL's effect on *k_c_* values in PC membranes are lacking, a more quantitative evaluation on PSM's effect in this case is difficult. However, its tendency to increase *K_A_* would suggest a role also on bending rigidity.

The fact that our values for the bilayer thickness agree with an AFM study, reporting a difference of 0.6–0.9 nm between *l_d_* and *l_o_* phases, is an indication that our model systems are in line with the experimental *l_o_*/*l_d_* phases [43]. However, when comparing the diffusion coefficients between systems *S_A_* to *S_C_,* we find that systems *S_A_* and *S_B_* are relatively much more slowed down than predicted from the changes of pure POPC bilayer upon addition of 25–30 mol% CHOL [33]. This further supports the idea that SM (together with CHOL) has an additional role in rigidifying the bilayer and consequently slowing down diffusion. For comparison, a recent pulsed-field gradient NMR study [44] reported two populations of *D* values in DOPC–SM–CHOL mixtures with 10–30 mol% CHOL at 300 K, one corresponding to *l_d_* phase (*D* ≈ 1 × 10^−7^ cm^2^/s) and the other to *l_o_* phase (*D* ≈ 1 × 10^−8^ cm^2^/s). As the exact lipid composition within the proposed domains is unknown, our simulated *D* values for systems *S_A_* and *S_B_* are in good agreement with the proposed *l_o_* phase. This is interesting, since the *l_o_* phase is usually characterized as having similar diffusion rates with the *l_d_* phase. Recent evidence on large variations in the properties of a single *l_o_* phase [45] also supports the idea that bilayers *S_A_* and *S_B_* do display the *l_o_* phase. Clearly, diffusion within raft domains is strongly suppressed due to the presence of PSM and CHOL.

The material properties of lipid bilayers have been suggested to play a major role in regulating the activity and partitioning of membrane proteins. First, the thickness difference of raft and nonraft membranes may be relevant due to the effects of hydrophobic matching [46,47]. For example, the free energy of opening of a bacterial stretch-activated channel has been observed to change from 4 to 20 *k*_B_ *T* when the acyl chain length of the surrounding PC-lipids changes from 16 to 20 carbons [48]. Another example is the transmembrane protein OmpA, whose free energy of unfolding was reported to change by about 5 *k*_B_ *T* per nm when the hydrophobic thickness of the surrounding saturated PC-membrane was varied [49]. Using this value as a simplistic estimate for the effect of hydrophobic thickness, one gets a difference of about 4 *k*_B_ *T* in the free energy of unfolding when this particular protein would be transferred from nonraft to raft membrane. As the higher bending rigidity of the raft membrane probably decreases the ability of the membrane to adapt its thickness to match the hydrophobic thickness of the protein, the actual value should be larger than the above estimate. The role of membrane elasticity in protein functionality is further emphasized by the fact that, based on recent studies, it costs much more energy to deform a membrane by changing its area per lipid than by bending or chain tilting [50]. It has been suggested that the free energy to create a protein-shaped cavity in a bilayer is proportional to *K_A_* [51], and evidence exists that the binding free energy of certain amphipathic peptides indeed depends linearly on *K_A_* [52]. Our data suggests a 5-fold to 14-fold difference in the values of *K_A_* between raft and nonraft membranes (see Table 1), which practically means a free energy cost of about 4–8 *k*_B_ *T* when a membrane protein (Mellitin) is transferred from a nonraft to a raft environment [52]. Summarizing, the elasticity of raft-like membranes is substantially different from that of nonraft membranes, and this likely influences membrane protein functionality.

### Lateral Heterogeneity

**Figure 2:**
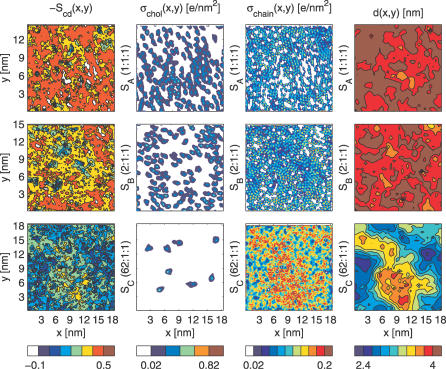
Snapshots Averaged over the Last 10 ns from the End of Each Simulation The deuterium order parameters, *S*_CD_ *,* of selected carbons (C5–C7) of POPC and PSM chains were binned in the xy-plane (column 1, from left). The in-plane electron densities, σ, have been plotted separately for CHOL (column 2) and the selected chain carbons (column 3). The average bilayer thickness, *d,* was obtained from the grid of the undulation analysis (column 4). Systems *S_A_* to *S_C_* are represented on rows from top to bottom, respectively. Only the bottom leaflet has been used for columns 1–3, whereas both leaflets were used for column 4. The equivalent plots for the top leaflet have been presented in Figure S2.

The above results highlight the different bulk properties of raft-like domains with respect to more disordered bilayers. However, as becomes evident below, raft domains are also characterized by strong spatial and temporal variations. Figure 2 reveals lateral heterogeneity in the calculated deuterium-order parameter values (*S*_CD_) when averaged over 10 ns. The nature of chain ordering varies in different systems. System *S_A_* exhibits the highest overall order (average *S*_CD_ = −0.41) that is almost uniformly distributed over the bilayer plane and broken only by a few small low-order areas and empty points due to poor sampling. System *S_B_* is slightly less ordered (*S*_CD_ = −0.36) and contains domains of a few nanometers in size, differing significantly in their *S*_CD_ values. The overall ordering in *S_C_* is much weaker (*S*_CD_ = −0.18) than in the two other systems, but even *S_C_* displays lateral heterogeneity, though the domains appear larger, smoother, and with smaller variations in the *S*_CD_ values. The average *S*_CD_ values are in line with corresponding experimental order parameter profiles of fluid POPC [53,54] and DPPC–CHOL mixtures with similar CHOL concentrations [45,55].

In Figure 2, the more ordered regions in *S*_CD_ plots are clearly correlated with a higher density of CHOL. This is in line with a previous study showing CHOL's ability to order the neighboring acyl chains within a radius of few nanometers [56]. The σ_chain_ plots in Figure 2 reveal high localization of the chains in *S_A_,* whereas in *S_B_* some of the regions are smeared out. The *S_C_* plot is much more homogeneous, indicating higher overall mobility and more isotropic distribution of the chains. In *S_C_,* the small concentration of CHOL does not seem sufficient to account for the observed large-scale lateral heterogeneity in chain-order parameters. Instead, we find that the *S*_CD_ value is clearly correlated with bilayer thickness. This is particularly supported by the fact that the amplitudes of the large-scale peristaltic wave modes are significantly larger for system *S_C_* than for the other systems (see Figure S6). Even though the autocorrelation functions for most of the largest undulations and peristaltic modes decay roughly within a few nanoseconds (unpublished data), some modes display much longer decay times. In particular for system *S_C_,* this may be related to the heterogeneity induced by the few CHOL and SM molecules that are embedded in the bilayer.

**Figure 3:**
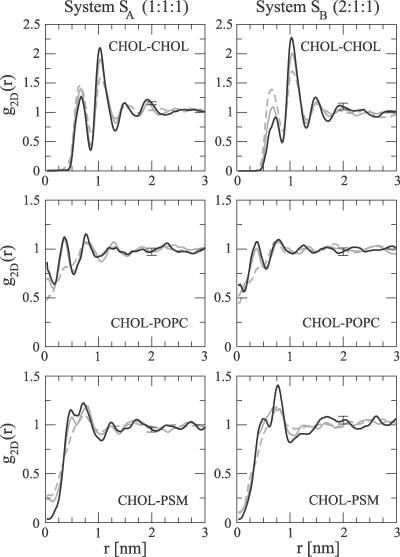
2-D Radial Distribution Functions between the Molecular Center of Mass Positions in *S_A_* and *S_B_* The figures show the time evolution in the system at three different time intervals: 0–10 ns (gray, dashed), 30–40 ns (gray), and 90–100 ns (black). The error bars for the black curve indicate the average difference of the two monolayers.

To judge our findings for lateral heterogeneity, it is worthwhile to stress the slow dynamics in the bilayer plane: despite the extensive time scale simulated, the lateral diffusion coefficients indicate that the molecules move in the plane of the membrane approximately over only their own size within the simulated time scale. Hence, it is evident that the simulation time is not long enough to adequately relax the large-scale structure of the initial configuration and lead to complete mixing of the lipids. The nanoscale heterogeneity observed in this work could thus be debated. However, there is reason to emphasize that while systems *S_A_* and *S_B_* were started from different initial configurations, they lead to similar conclusions. Further, the small-scale movements of the molecules relative to each other can be characterized; see the 2-D radial distribution functions in Figure 3. The unfavorable close contacts of CHOL–CHOL pairs are revealed by the lowering of the nearest neighbor peak in time. Simultaneously, the secondary peak at 1.0 nm increases, indicating small-scale reorganization of CHOL molecules. Significant changes in time can also be seen in the other plots of Figure 3, revealing the tendency of closer contacts between CHOL–POPC center of mass pairs with respect to PSM–CHOL pairs. In all, this provides further support for lateral reorganization and heterogeneity. The details of the lipid–lipid interactions are related to the widely speculated specific interaction between SM and CHOL, which is discussed elsewhere [57,58].

### Lateral Pressure Profiles

**Figure 4:**
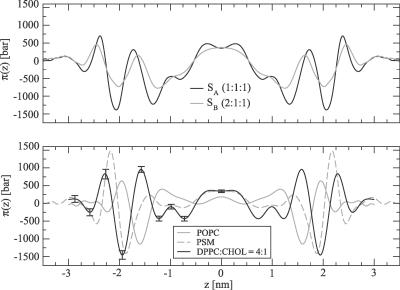
Lateral Pressure Profiles of Systems *S_A_* and *S_B_* (Top) and of Previously Simulated Pure POPC/PSM Systems and a Binary DPPC–CHOL System (Bottom) The center of the membrane is at *z = 0*. The graphs have been averaged to be symmetric on both sides of the center and smoothed by adaptive high-order spline fitting [90]. Error bars are statistical errors for each slab. The errors have been shown for only one of the monolayers of the DPPC–CHOL system because they are equal for both monolayers and also smaller or equal for the other systems.

Structure and dynamics of membrane proteins are likely to be influenced by the lateral pressure profile, which has been proposed as a mechanism for, e.g., general anesthesia [20,59]. To elucidate this issue, we computed the lateral pressure profiles of various lipid membrane systems (see Figure 4). For a discussion of the coupling of the peaks in the lateral pressure profile with the molecular groups and different interaction types, see previous related simulation studies [19,60–63]. Here, we focus on a more generic issue, that is, the joint effect of CHOL and PSM on the pressure profile.

The pressure profiles across the membranes of *S_A_* and *S_B_,* shown in Figure 4A, indicate a striking difference compared with profiles in nonraft membranes (see Figure 4B): raft bilayers display qualitatively different behavior with a greater number of peaks as compared with single component POPC and PSM bilayers in *l_d_* phase. Rather, raft systems display a qualitative similarity to the DPPC–CHOL system, shown in Figure 4B. These observations are in line with previous simulation studies, if reports on other single component *l_d_* bilayers [60–62] are compared with binary PC–CHOL systems [19,63]. A remarkable difference found here is the significant increase of positive (repulsive) pressure at the middle of raft bilayers compared with pure POPC, the effect being particularly large in the case of raft-mixture *S_A_*.

Notably, the peak heights in the lateral pressure profile are of the order of 1,000 bar. Thus, molecules such as integral membrane proteins are under the influence of huge local pressures that likely affect their conformational state. Particularly, proteins whose cross-sectional area undergoes significant anisotropic changes when shifting from active to inactive state are likely to be governed or regulated by the pressure profile [20,64]. To further quantify this idea, we estimated the lateral pressure profile–induced component of the energy between open and closed conformations of a channel protein MscL (see Methods). For this quantity, we get Δ*W* = (11 ± 2) *k*_B_ *T* and (4 ± 1) *k*_B_ *T* for systems *S_A_* and *S_B_,* respectively. These are significantly higher than the values found for the pure POPC bilayer (1.9 ± 0.2) *k*_B_ *T*, the pure PSM bilayer (1.0 ± 0.6) *k*_B_ *T*, or the binary DPPC–CHOL bilayer (1.0 ± 0.4) *k*_B_ *T*. The above result for a POPC bilayer is in agreement with the previous calculation by Gullingsrud and Schulten [62], who found 1.7 *k*_B_ *T* for POPC. The positive values of Δ*W* indicate that the lateral pressure profiles of these bilayers lower the open state energy of MscL relative to the closed state; that is, they are in favor of the open state. Especially interesting are the large values found for the raft systems, which suggest that the lateral pressure profiles characteristic of raft-like environments would facilitate the opening of MscL. For comparison, it has been estimated that the free energy difference associated with the opening of MscL is about 20–50 *k*_B_ *T* [62,65]. The contribution due to the pressure profile in a raft domain could therefore be significant. In general terms, it is clear from the above estimates that the equilibrium probability of MscL to be in the open state must be significantly altered by the pressure profile of the lipid environment. Additionally, we wish to underline that the values for Δ*W* have been estimated for different bilayers that are all under identical surface tension conditions (γ = 0) and are thus not related to the usual picture of the effect of overall stress on mechanosensitive channels.

## Discussion

In this work, we have elucidated properties of lipid raft mixtures through atom-scale simulations and compared them with properties of a bilayer in liquid disordered phase. We found that the presence of PSM and CHOL in *S_A_* and *S_B_* not only significantly enhanced the lateral packing of lipids and increased the acyl chain order, but also reduced the lateral diffusion rates by more than an order of magnitude when compared with the *l_d_* phase. This observation is contradictory to the traditional definition of the *l_o_* phase, but is in agreement with recent reports on varying properties of this particular phase [45,54]. It is interesting to note that the difference in the lipid dynamics of the different phases may in itself have a contribution to the dynamical partitioning of membrane proteins [5], as they spend more time in the ordered domains due to slower diffusion and allow more time for cross-linking between proteins to occur.

The elasticity of the raft mixtures was found to be reduced significantly when compared with *S_C_*. The fact that this reduction was greater than expected from previous reports on binary PC–CHOL mixtures, suggests that SM has a further rigidifying effect on raft mixtures. The 5-fold to 14-fold increase in *K_A_* suggests significant implications on the partitioning of membrane proteins. First, the free energy of creating a cavity to the membrane, and thus the solvation free energy of a protein into a membrane, is directly proportional to *K_A_,* which leads to unfavored partitioning of certain proteins into raft-like membranes. On the basis of a recent experimental report [52], we estimated that the transfer free energy of Mellitin from nonraft to raft membrane would be about 4–8 *k*_B_ *T*. Second, the difference in thickness of about 0.8–0.9 nm between raft and nonraft membranes suggests a contribution to the transfer free energy of proteins due to changed hydrophobic matching. This effect is practically always present and the reported strength of the effect, about 5 *k*_B_ *T* per nm [49], makes it comparable to the effect of the *K_A_*.

The lateral heterogeneity in the simulated membranes was found to be related to either the tendency of CHOL to order neighboring acyl chains or to the relatively slow peristaltic modes of the bilayer. The emergence of these heterogeneities may be related to the idea of small granular arrangement of nanodomains in biological membranes [9,12]. Considering the perhaps surprisingly slow diffusion rates observed for the *l_o_* phase of the ternary mixtures in this study, it suggests an absolute minimum of about 10–100 ns for the lifetimes of the domains. Also, the analysis of the heterogeneity provided more support for the idea that CHOL changes the lipid environment in its local neighborhood, e.g., by increasing the order of the acyl chains.

Analysis of the raft-like membranes *S_A_* and *S_B_* revealed large differences in lateral pressure profiles when compared with bilayers in *l_d_* phase, but also changes of significant magnitude in the local pressure were found in comparison with PC–CHOL systems. All membrane proteins, which undergo anisotropic structural changes between functional states, are likely to be affected by the lateral pressure profile. A good example would be proteins that tilt their helices when opening a channel, such as the MscL [66]. We found that the free energy difference between the open and closed states of the MscL channel changed from 1.0 *k*_B_ *T* to 4–11 *k*_B_ *T* when single component bilayers in *l_d_* phase were compared with raft mixtures. This result, together with previous reports on pressure profiles of similar systems [19,63], provides strong evidence for the idea that the lipid environment plays an important role in regulating the activity of certain membrane proteins through changes in lateral pressure profile. Though only a few experimental studies have been done to assess local pressures within the bilayer [26,67], evidence exists that the activity of a number of membrane proteins is dependent on the lipid composition and thus very probably on the lateral pressure profile [67,68]. For example, the free energy of binding of alamethicin has been reported to be a simple function of monolayer spontaneous curvature [69], which is most probably related to changes in local pressures.

The role of the lipid environment has been discussed in relation to a variety of membrane proteins, from mechanosensitive channels such as MscL [48,62,70] to other important channels such as rhodopsin [71], KcsA [67], P-glycoprotein [72,73], the insulin receptor [17], and others whose activity has been shown to depend on the membrane composition [67]. It has been shown that different lipids have different binding affinities on the surface of membrane proteins [74] and that the specific lipid-protein interactions probably play a role in regulating the activity and/or partitioning of certain proteins such as the yeast cytochrome *bc_1_* complex [75]. However, various evidence exists that generic interaction mechanisms in terms of for example elastic properties of the membrane are also important for a number of membrane proteins [47]. For example, different sterols have been shown to alter the elastic properties of membranes in a similar manner, if only applied in different concentrations [76]. Finally, it is exciting to note that the present results also provide support for a recent suggestion that the (unknown) mechanism of general anesthesia is related to changes in the lateral pressure profile due to incorporation of anesthetics, such as alcohols, into the membrane [20,64,77].

From now on, the quest is to understand the (concerted) effect of different lipid species on the lateral pressure profiles and the interplay between lipid environment and protein activity. The lateral pressure profile is an important quantity, as many membrane elastic coefficients (such as bending modulus, spontaneous curvature, and the saddle splay modulus) can be directly extracted from it [78]. In the future, it would be highly useful to see computational works on lipid bilayers that gather enough statistics to evaluate the relationship between these quantities and to increase our understanding of their relationship. Also, it would be highly interesting to develop experimental techniques to measure the pressure profiles and to relate these to the already existing simulation data of different membrane compositions.

## Materials and Methods

Starting coordinates were obtained by expanding a previously equilibrated POPC bilayer [28] to 1,024 lipids. Two ternary mixtures were created by replacing random POPC molecules by PSM and CHOL to result in POPC:PSM:CHOL = 1:1:1 or 2:1:1 molar ratios (systems *S_A_* and *S_B_,* respectively), whereas for the third system (*S_C_*) we replaced 32 selected POPC molecules to result in a POPC matrix with eight CHOL-PSM dimers and 16 monomers that are as far as possible from each other. The configuration in *S_C_* was created to study the local interactions between PSM and CHOL in a POPC matrix, which will be discussed elsewhere [58]. The force-field parameters for POPC [79], PSM [34], and CHOL [80] were obtained from previous works. Each of the three bilayers were fully hydrated with about 28 SPC (simple point charge) water molecules/lipid [81], resulting in ∼140,000 atoms per system (see Figure 1). Using GROMACS (http://www.gromacs.org) [82] for integrating the equations of motion with a 2-fs time step, each system was initially equilibrated by the Langevin thermostat in NVT-ensemble (50 ps) and then in NpT-ensemble (500 ps). The first 5 ns of the actual simulations were run in NpT-ensemble (*T* = 310 K, *p* = 1 atm) using the Berendsen thermostat and barostat [83], after which we switched to the Nosé-Hoover thermostat and the Parrinello–Rahman barostat to produce the correct ensemble. The pressure coupling was applied in a semi-isotropic way to result in zero surface tension. The long-range electrostatic interactions were accounted for by the reaction-field method (with *r_c_* = 2.0 nm) and a 1.0-nm cutoff was used for the Lennard–Jones interactions. Reaction-field has been shown to be a reliable and well-scalable method for simulating noncharged lipid bilayers [84]. The simulation time was 100 ns for *S_A_* and *S_B_,* but 50 ns for system *S_C_,* which together took about ten cpu-years on a parallel machine. For the analysis, we have included the last 40 ns of each simulation trajectory whenever not indicated otherwise.

The equilibration of the bilayer structure was monitored by the area per lipid (see Figure S1). The magnitude of area fluctuations were used to estimate the area compressibility of each bilayer [85]. The average bilayer thickness was estimated from the peak-to-peak distance of the electron density plot of all atoms across the simulation box. To characterize undulatory and peristaltic motions, we followed the procedure by Lindahl and Edholm [30], in which a grid was fitted to selected atoms in the POPC and PSM backbone (glycerol C2 in POPC and the corresponding carbon in PSM). The grids for the two monolayers were then averaged for undulatory analysis whereas their difference was used for describing the peristaltic motions, and in both cases 2-D FFT was applied to the grid points. *k_c_* was estimated by summing over the undulatory spectral modes and utilizing the formula <u^2^_und_ > ≈ *k*_B_ *TA*/(8.3π^3^ *k_c_*). Consistent results for *k_c_* were found through a fit to the function u^2^_und_ (k) ∼ *k* ^−4^.

The deuterium (NMR)–order parameter *S*_CD_ values were calculated from the diagonal elements of the molecular order tensor (see [84]) at selected carbon locations of the PSM and POPC chains. To characterize the lateral heterogeneity in the system, carbons 5–7 were chosen from each acyl chain (together with the structurally correspondent carbons from the sphingosine chain), and the instantaneous *S*_CD_ values were binned on a grid on the bilayer plane. Similarly, the average in-plane electron densities were calculated by binning the number of electrons in the selected molecules or atoms. For the 2-D radial distribution functions, *g*_2D_(*r*), we used the projected center of mass positions of the lipid molecules. The centers of mass were also used to obtain the lateral diffusion coefficients (for details see [15]).

Finally, lateral pressure profiles were determined using an approach similar to the ones presented and validated by several authors [19,60–62], more details of our method in [86]. The lateral pressure was calculated using the Irving–Kirkwood contour and dividing the systems in ∼0.1 nm thick slabs (100 slabs). Pairwise forces were calculated from the force field description and MD trajectory. A 2.0-nm truncation was used for electrostatic interactions. Constrained forces arising from SETTLE and LINCS were calculated from the general equation by Hess et al. [87]. As undulations in system *S_C_* render the lateral pressure calculation more difficult, we chose three previous simulations on single-component lipid systems, POPC [86], PSM [34], and a binary 1:4 DPPC–CHOL [17] for reference. For each system, the pressure profile was calculated the same way. To estimate the effect of pressure profile on membrane proteins, we followed the approach introduced by Cantor [88] and later applied to molecular simulation data of single-component bilayers by Gullingsrud et al. [62]. As a model we use the mechanosensitive ion channel MscL, whose conformation has been found to change anisotropically between cylindrical (open) and cone (closed) shapes [89]. Here we calculate the work, Δ*W,* done against the lateral pressure profile to alter the shape of the membrane cavity occupied by the protein as it changes conformation from the closed to an open state. Then Δ*W* can be written as:

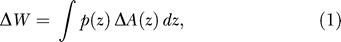
where Δ*A*(*z*) is the change in the cross-sectional area of the protein and *p*(*z*) is the pressure profile. Here, we use an approach identical to that used in [62], and identical values for Δ*A*(*z*) for MscL as used in [62], in which the area is kept unchanged in the middle of the membrane between the two states. Error bars for Δ*W* have been calculated using results for different monolayers. It is, however, important to realize that Δ*W* depends on the second moment of the lateral pressure profile [62] and thus is susceptible to small changes of lateral pressure far from the bilayer center. Therefore, extra caution must be followed when interpreting these results. Also, in this approach the influence of inserting a protein into the membrane on the lateral pressure profile is not taken into account.

## Supporting Information

Figure S1 The Area per Lipid versus Simulation Time (509 KB EPS)

Figure S2 Averaged Snapshots from the Last 10 ns of Each Simulation The data is represented as in Figure 2 of the main article, but plotted for the top monolayer (columns 1–3) instead of the bottom monolayer.
(212 KB EPS)

Figure S3 Snapshots (1-ns Averages) Revealing the In-Plane Electron Density of CHOL at 10-ns Time Intervals Columns A1–C1 are the bottom monolayer and columns A2–C2 the top monolayer in systems *S_A_* to *S_C_,* respectively.
(1.2 MB EPS)

Figure S4 Snapshots (1-ns Averages) Revealing the Undulation and Peristaltic Motions at 10-ns Time Intervals Columns A1–C1 are the average bilayer height (*z*(*x;y*), the mean height of the two monolayers), whereas columns A2–C2 are the bilayer thickness (*d*(*x;y*), the difference in height of the two monolayers) in systems *S_A_* to *S_C_,* respectively. For calculating *z*(*x;y*) and *d*(*x;y*), we used the grid method discussed in the Methods section.
(725 KB EPS)

Figure S5 Undulatory Spectral Intensity per Wave Mode versus Wave Vector Magnitude for Systems *S_A_* to *S_C_*
The legend shows *k*_c_ values calculated by two different methods, the summing method utilizing Equation 4 and fitting Equation 3 in [30].
(183 KB EPS)

Figure S6 Peristaltic Spectral Intensity per Wave Mode versus Wave Vector Magnitude for Systems *S_A_* to *S_C_*
(97 KB EPS)

## Abbreviations

A: average area per lipid
CHOL: cholesterol
d: bilayer thickness
*D*: lateral diffusion coefficient
K_A_: area compressibility modulus
k_B_T: thermal energy scale (k_B_ is the Boltzmann constant, T is the absolute temperature)
k_c_: bending rigidity
l_d_: liquid disordered phase
l_o_: liquid ordered phase
PC: phosphatidylcholine
POPC: palmitoyloleoylphosphatidylcholine
PSM: palmitoylsphingomyelin
S_CD_: deuterium order parameter values
SM: sphingomyelin
